# Drumming with BEAT: a pilot study on the impact of drum set playing on motor control and quality of life in Parkinson's disease

**DOI:** 10.3389/fresc.2026.1671559

**Published:** 2026-02-09

**Authors:** Min-ji Kim, Soo Ji Kim

**Affiliations:** 1 Department of Neurorehabilitation, National Rehabilitation Center, Seoul, Republic of Korea; 2 Music Therapy Education, Graduate School of Education, Ewha Womans University, Seoul, Republic of Korea

**Keywords:** drum set playing, motor control, music therapy, Parkinson's disease, quality of life

## Abstract

Various interventions have been proposed to improve motor and non-motor symptoms in patients with Parkinson's disease (PD). Rhythm-based music interventions have the advantage of addressing multifaceted rehabilitation needs, especially those that use set drumming to induce structural coordination of the limbs, which can provide motor, cognitive, and psycho-emotional benefits. This pilot case series examined the feasibility and preliminary outcomes of a seated drum set intervention—referred to as the BEAT program (Bilateral Engagement through Active Drumming in Music Therapy) in three community-dwelling adults with idiopathic PD (Hoehn and Yahr stages 2–3). Participants completed ten 30-minute sessions over 6 weeks using an acoustic drum set programmed with structured rhythmic exercises that progressively increased in complexity. Pre/post assessments included the Berg Balance Scale (BBS), Timed Up and Go (TUG), 9-Hole Peg Test (9HPT), Box and Block Test (BBT), MIDI-based drum tapping synchronization, Trail Making Test (TMT A/B), Korean Color Word Stroop Test (K-CWST), Parkinson's Disease Questionnaire-39 (PDQ-39), and Korean Geriatric Depression Scale (K-GDS). Semi-structured interviews were also conducted. Across the three cases, participants showed generally favorable changes in balance and upper limb dexterity, along with selected improvements in cognitive test performance. In contrast, depression and quality-of-life scores showed mixed patterns, despite consistently positive accounts of motivation, enjoyment, and perceived functional benefits in the interviews. These preliminary findings suggest that a therapeutic approach to drum set playing may be a feasible and individually adaptable rehabilitative strategy for people living with PD, warranting further evaluation in larger, controlled trials.

## 1 Introduction

Parkinson's disease (PD) is a chronic, progressive neurodegenerative disorder primarily associated with the loss of dopaminergic neurons in the substantia nigra, a key region of the midbrain involved in motor control (1). The resulting dysfunction in basal ganglia circuitry leads to a range of motor symptoms, including bradykinesia, rigidity, resting tremor, and postural instability. In addition to these motor impairments, many individuals with PD also experience non-motor symptoms such as cognitive decline, mood disturbances, and autonomic dysfunction, which further affect daily functioning and quality of life. The degeneration of motor pathways compromises the initiation, regulation, and coordination of voluntary movements, making it difficult for patients to execute smooth, goal-directed actions (2). These deficits can hinder the performance of routine tasks and significantly reduce independent living.

Difficulties in interlimb coordination, evident even in the early stages of the disease, affects both leg and arm coordination (3–5). Such deficits in coordination contribute to challenges in spatial and temporal alignment, thereby impacting daily independence and quality of life (6). The necessity of motor control for body movement and stability cannot be overstated; it involves a sophisticated process of regulating and coordinating actions (7). From a dynamic systems theory perspective, the human body is seen as a complex, interconnected system where components like joints and neuromuscular elements must work in harmony to achieve movement goals (8). This theory underscores the importance of adapting limb coordination patterns to various conditions and task characteristics to optimize motor performance (9).

Current research based on dynamic systems theory indicates considerable variability in performance when external cues are used to enhance arm coordination in PD patients, especially in group settings or in the early stages of the disease (10, 11). Moreover, accumulating evidence suggests that individuals with PD benefit from interventions that require them to plan and adapt movements within changing spatial and temporal contexts, as demonstrated in upper limb coordination and functional training programs (11–13).

Among various exercise intervention approaches, rhythm-based interventions involve structured musical activities that rely on rhythmic cues and external auditory stimuli to improve the temporal organization of movement (14–16). These interventions have been shown to enhance motor coordination and movement speed, while also offering added benefits such as increased intrinsic motivation and improved emotional well-being (17, 18). Additionally, recent systematic reviews consistently report positive effects of rhythm-based interventions on gait, balance, and psychosocial functioning in PD rehabilitation, providing additional support for their clinical value (19–22).

A drum set consists of various percussion instruments and requires harmonious movement of both the upper and lower limbs, which distinguishes it from other instruments used in therapeutic interventions. It necessitates dynamic movement and intricate bodily coordination and control (23, 24). The diverse arrangement of drum set components demands precise adjustments in wrist, ankle, leg, foot, arm, and finger movements to manage the rebound of sticks upon contact with the instruments (25, 26). Differences in the placement and distances between instruments affect joint and muscle coordination required for movement angles, speeds, and intensity changes, thereby influencing overall bodily coordination (24). Additionally, playing the drum set requires anticipation of timing within musical structures and planning for subsequent movements, engaging both physical and cognitive resources (15, 24, 26). As such, drum sets have promising potential as a treatment modality that can simultaneously utilize various functional areas such as motor control and cognition.

Existing upper limb interventions for PD patients are often designed around repetitive, segmental movements, which may limit their functional applicability given that everyday activities require more complex, multi-joint actions (12, 13). In terms of functional relevance and encouraging active participation, playing the drum set can be an appropriate alternative. Drum set playing, which inherently requires advanced interlimb coordination and the integration of bilateral upper and lower limb movements, represents a music-based intervention that aligns with the motor and psychosocial challenges commonly observed in PD. Drum set playing can promote the expansion of music intervention strategies as a method that encourages active participation while appropriately using the upper and lower limbs. As a rhythmic activity that requires whole-body movement, it can help improve function and quality of life when tailored to the abilities and needs of PD.

Therefore, this pilot study developed and applied the BEAT program (Bilateral Engagement through Active Drumming in Music Therapy), a structured drum set-based intervention grounded in dynamic systems theory, to promote motor and cognitive functions in individuals with PD. The BEAT program was specifically designed to facilitate bilateral limb engagement through progressively challenging drumming tasks, thereby enhancing sensorimotor integration and active participation. The intervention was implemented with PD patients to examine its effects on functional improvement as well as psychological and emotional responses. Based on the analysis results, preliminary guidance for the integration of this music therapy approach into clinical practice was provided.

## 2 Methods

### 2.1 Criteria for participation

Participants were eligible for the study if they met the following criteria: (1) a confirmed diagnosis of PD by a neurologist; (2) age 50 years or older (to target late-onset PD and minimize heterogeneity) The lower age limit of 50 years was chosen to target late-onset Parkinson's disease and to minimize heterogeneity related to early-onset forms, which are typically defined as onset before age 50 years (27); (3) a score of 36 or lower on the Unified Parkinson's Disease Rating Scale (UPDRS-II); (4) a score of 23 or higher on the Korean Mini-Mental State Examination (K-MMSE), indicating sufficient cognitive function; (5) no uncorrectable visual impairments; and (6) no significant auditory limitations that would affect their ability to engage with musical components. Exclusion criteria were: (1) diagnosis of other neurological disorders (e.g., stroke, dementia); (2) severe cognitive impairment that would interfere with understanding instructions or participating in the intervention; (3) severe uncorrected visual or hearing impairments; (4) musculoskeletal conditions that prevented safe seated drum-set playing; and (5) unstable medical status or recent changes in antiparkinsonian medication within the past 3 months.

Recruitment occurred through advertisements on online PD community forums and at rehabilitation hospitals in Seoul, with ethical approval (IRB No. 4-2012-0483). Each participant provided informed consent after receiving a thorough explanation of the study's aims and procedures.

### 2.2 Participants' demographic characteristics

The demographic details and baseline clinical profiles of the participants are summarized in Table 1.

**Table 1:** Participants’ demographic information and pre-test results.

Characteristic	Participant A	Participant B	Participant C
Age (years)	86	67	74
Gender	M	F	F
Duration of illness (years)	8	13	13
Affected side	R	L	R
UPDRS-II (/52)	12	16	12
MMSE-K (/30)	23	28	28

#### 2.2.1 Participant A

Participant A was an 86-year-old male diagnosed with PD in 2015. He was able to walk independently without assistive devices but exhibited narrow steps and difficulty maintaining upper body posture and arm stability while walking. While seated, he leaned to the right, making it difficult for him to maintain trunk stability. Tremors were observed in his right hand at rest, but he did not have difficulty writing with a pen. According to the participant, he performed most daily activities independently but required assistance when standing up from a chair or descending stairs and experienced difficulty balancing while walking. During the interview, Participant A repeatedly expressed concerns about his condition. He stated he enjoyed listening to music in his daily life and used to enjoy singing when he was younger.

#### 2.2.2 Participant B

Participant B was a 67-year-old female diagnosed with PD in 2010. She exhibited a gait pattern in which her right foot deviated outward while walking, and she leaned to the left while seated. Tremors and rigidity were observed in her left hand when writing or using a mobile phone. According to the participant, she could independently perform daily activities and used public transportation, but she recently experienced difficulties in balancing. She also reported tremors and rigidity in her hands while performing movements in daily life. During the interview, Participant B initially appeared cheerful, but tears welled up as she talked about her past time before the onset. She expressed interest in learning drums but was worried about it being challenging for her. She reported that when she wanted to listen to music, she would play the radio but that she did not do this frequently.

#### 2.2.3 Participant C

Participant C was a 74-year-old female diagnosed with PD in 2010. She exhibited decreased upper body flexion and arm tremors, with tremors observed in her hands and feet at rest. According to the participant, she could independently perform daily activities and used public transportation. However, she experienced difficulties with balance and expressed fear while walking and using stairs in daily life. She also reported recent difficulties standing up from a chair due to back pain. During the interview, Participant C began the conversation with a bright expression and cheerful voice. However, tears welled up as she recounted her past, when, despite being healthy, she was unable to learn to play an instrument due to the need to earn a living.

### 2.3 Research design and procedure

The intervention was conducted from October 12 to December 3, 2022, with sessions administered sequentially according to each participant's enrollment schedule. This study employed a multiple case design involving three individuals diagnosed with idiopathic Parkinson's disease. As an exploratory investigation, formal sample size estimation was not performed, and no interim analyses or stopping rules were applied. The study aimed to assess the potential effects of drum set playing on motor control in individuals with PD through observation of individual cases, with the goal of generating preliminary data to inform future controlled studies. Accordingly, the primary aim of this pilot case series was to explore the feasibility and acceptability of the BEAT drum set program for individuals with PD and to describe preliminary changes in motor control, cognitive performance, and quality of life.

To ensure the validity of the intervention's theoretical basis and clinical applicability, the intervention was verified by three professional music therapists who each had more than 3 years of professional experience. Three music therapists independently assessed the appropriateness of the intervention using a 5-point Likert scale. The evaluation criteria included the alignment of the intervention with therapeutic goals and its suitability for implementation by individuals with PD. The scores provided by the three therapists were aggregated, and an average score was calculated, yielding an intervention validity rate of 81%. Informed consent forms were signed by the participants after providing them with information about the study. After obtaining informed consent, the pretest was conducted.

The intervention began within 1 week of obtaining informed consent. Posttest measures and post interviews were conducted within 5 days after the final session of the intervention. The intervention was planned to be conducted once or twice a week for a total of 10 sessions over 6 weeks to accommodate the participants’ schedules, with each session lasting 30 min. These sessions were conducted in a one-on-one format in a dedicated space in a music therapy clinic in Seoul.

### 2.4 Outcome measures

#### 2.4.1 Motor outcome measures

To evaluate lower limb coordination, balance assessments were conducted using Timed Up and Go (TUG) and the Berg Balance Scale (BBS). The Timed Up and Go (TUG) test measures functional mobility, balance, and fall risk by timing how long it takes a person to stand up from a chair, walk 3 m, turn around, and sit back down (28). The TUG has demonstrated good to excellent retest reliability (*r* = 0.73–0.99) and construct validity as a measure of functional mobility and fall risk in individuals with PD (29). The Berg Balance Scale (BBS) assesses balance and fall risk through 14 functional tasks scored on a 0–4 scale, commonly used for elderly and neurological patients (30). The Korean version of the BBS has shown excellent interrater reliability (ICC = 0.97) and intra-rater reliability (ICC = 0.95–0.97) in stroke patients, supporting its use as a reliable balance measure in clinical populations (31). To assess participants' hand function, the Box and Block Test (BBT) and the Nine-Hole Peg Test (9-HPT) were performed. The Box and Block Test (BBT) evaluates hand coordination, speed, and accuracy by measuring how many blocks a person can move with one hand in 60 s (32). The BBT has demonstrated excellent test–retest reliability (*r* = 0.93–0.98) and interrater reliability (ICC = 0.95–0.99), as well as strong convergent validity with other upper limb function measures in individuals with stroke and other neurological conditions (33). The Nine-Hole Peg Test (9-HPT) assesses fine motor coordination and dexterity by timing how quickly a person can place and remove nine pegs from holes (34). The 9-HPT has demonstrated excellent test–retest reliability (ICC = 0.88–0.91) in individuals with PD (35).

To measure upper limb motor control, a MIDI-tapping test was conducted by connecting the ALESIS electronic drum pad to the Cubase-MIDI program. The participant performs a task using mallets in both hands to play an electronic drum pad, either simultaneously or alternately, in response to auditory cues. The auditory cues are provided at different tempo conditions for these two types of task performances. At the start of the performance, the participant plays at a comfortable tempo, which is recorded as the baseline tempo. Subsequently, auditory cues are provided at 10% and 20% faster or slower than this baseline tempo, and the participant adjusts their performance accordingly. The closer this average value is to 0, the more likely it is that the movement was planned and performed accurately according to external stimulation (36, 37). Previous studies have shown that similar MIDI-based timing tasks exhibit consistent performance patterns across repeated measurements and sensitivity to temporal synchronization, suggesting that they can serve as reliable and valid indicators for detecting changes in upper limb motor control following rhythm-based interventions (36).

#### 2.4.2 Cognitive outcome measures

To assess attention and frontal lobe function, cognitive evaluations were conducted using the Trail Making Test (TMT) and the Korean-Color Word Stroop Test (K-CWST). The TMT consists of Parts A and B, evaluating divided attention, attentional control, and frontal lobe function (38). The TMT has demonstrated acceptable test–retest reliability and strong construct validity as a measure of processing speed and executive function, with performance closely linked to task switching, working memory, and inhibition (39). The K-CWST presents two cards with the words “red,” “black,” “blue,” and “yellow” written in four colors. Participants are asked to read the words (Word Reading) and name the colors (Color Reading) as quickly as possible. The time taken to read 112 words and the number of errors were measured and evaluated (40). Korean Stroop measures, including the K-CWST, have shown good internal consistency (Cronbach's *α* = 0.78) and strong convergent validity with other Korean Stroop indices (*r* = 0.81), supporting their reliability and validity for assessing inhibitory control and frontal lobe function in older adults (41).

#### 2.4.3 Psychosocial outcome measures

To evaluate participants’ depression and quality of life, we used the Korean version of the Geriatric Depression Scale (K-GDS) and the Parkinson's Disease Questionnaire (PDQ-39). The K-GDS is a self-report instrument designed to assess depression in older adults, focusing on emotional and psychological symptoms (42). The K-GDS has demonstrated good internal consistency (Cronbach's *α* = 0.88), excellent test–retest reliability (*r* = 0.91), and significant correlations with other depression and anxiety scales (*r* = 0.56–0.63), indicating robust reliability and convergent validity in Korean older adults (43). The PDQ-39 assesses the health-related quality of life in patients with PD across eight domains, including mobility and emotional well-being (44). The PDQ-39 has demonstrated satisfactory internal consistency for its domain scores (Cronbach's *α* = 0.58–0.80; *α* = 0.91 for the summary index) and excellent split-half reliability (Guttman coefficient = 0.72–0.94), as well as significant correlations with motor and non-motor symptom scales, supporting its reliability and construct validity for assessing health-related quality of life in Korean patients with PD (45).

#### 2.4.4 Post-intervention interview

To enrich the analysis of the research results, semi-structured interviews were conducted within 5 days after the final session, in addition to quantitative data. These interviews were conducted to explore what the participants experienced during the intervention period, including functional changes and personal meanings. All interviews were audio-recorded, transcribed verbatim, and coded by theme. To enhance the credibility of interpretation and the rigor of analysis, the coded transcripts were independently reviewed by external evaluators.

### 2.5 Drum set-based music therapy

#### 2.5.1 Drum set

**Figure 1:**
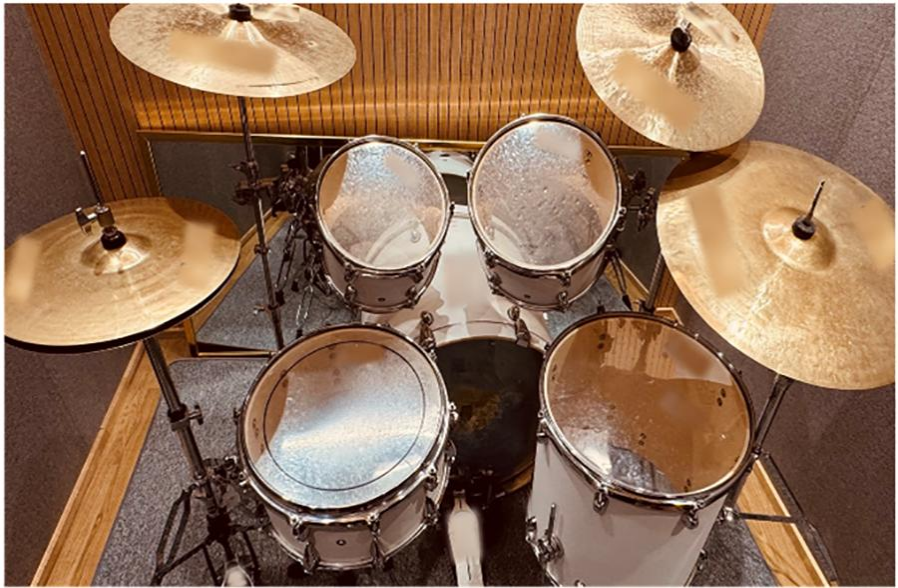
Drum set configuration for the intervention.

An acoustic drum set was used as the intervention instrument for improving motor control. The acoustic drum set consisted of the following drums and cymbals: snare drum, bass drum, high-tom, middle-tom, floor-tom, hi-hat cymbals, crash cymbals, and ride cymbals (Figure 1 for reference). During the intervention, the music therapist could use an acoustic guitar (Martin DJR-10E Streetmaster) or a keyboard (KORG-SP250) to provide music for the interactive music playing segment of each session during which the researcher (music therapist) and participant played music together. All sessions were conducted individually.

#### 2.5.2 BEAT intervention

The BEAT intervention used in this study was conceptualized as a neurologic music therapy protocol targeting motor control impairments in individuals with PD. For each session, three graded activity goals corresponding to stages 2–4 in Table 2 were planned. Each activity was structured with varying levels of difficulty according to specific musical elements (e.g., tempo, meter, dynamics) and the individual functional level of each participant (46; Table 2 for reference).

**Table 2:** BEAT program intervention.

Stage	Main activity	Goal		Playing tasks	Musical elements
1	Limb stretching	Inducing physical relaxation		Stretching and relaxation of the upper and lower limbs	-
2	Separate playing	Intralimb coordination	Upper limbs	- Playing simultaneously or alternately with both hands	Accent, crescendo, decrescendo tempo
			Lower limbs	- Playing simultaneously or alternately with both feet	
3	Combined playing	Interlimb coordination	Self-paced (tempo)	- Playing simultaneous or alternating of upper and lower limbs	Beat, tempo, meter
			Paced Change (−20% to 20%)	- Modification of Playing Positions by Utilizing the drum set Structure	
4	Rhythmic playing	Continuous limb coordination		Playing with comprehensively patterned rhythms used in the session	Accent, crescendo, decrescendobeat, tempo, meter
5	Interactive playing with a music therapist	Enhancing positive emotional experience		Playing the presented music with a music therapist or performing improvisation	

This drum set playing intervention consisted of (1) preparatory limb stretching, (2) individual playing, and (3) interactive playing with a music therapist. To prepare the musculoskeletal system and reduce muscle tension in the neck, shoulders, wrists, and ankles, the music therapist provided patterned musical cues that participants were instructed to imitate. Individual playing was then carried out in three stages: separate playing for intralimb coordination, combined playing for interlimb coordination, and rhythmic playing for more continuous interlimb coordination.

In the intralimb coordination phase of the intervention, upper and lower limbs were engaged in isolated playing tasks to facilitate targeted motor activation of each limb segment. For upper limb exercises, participants employed a matched grip to hold a drumstick in each hand and performed alternating patterns on the snare drum, high-tom, middle-tom, and floor-tom. These movements were intended to promote distal motor control by regulating wrist flexion and extension using the pronator and supinator muscles of the forearm. Musical elements such as accents and crescendos were incorporated to shape dynamic changes and enhance proprioceptive awareness during these movements. For the lower limbs, heel-down and heel-up techniques were used to operate the bass drum and hi-hat pedal. These movements activate flexion and extension of the ankle and hip joints, as well as major muscle groups including the biceps femoris and tibialis anterior. Repeated use of the pedals was intended to challenge lower limb stability and ankle joint range of motion, within the context of structured rhythmic tasks.

**Figure 2:**
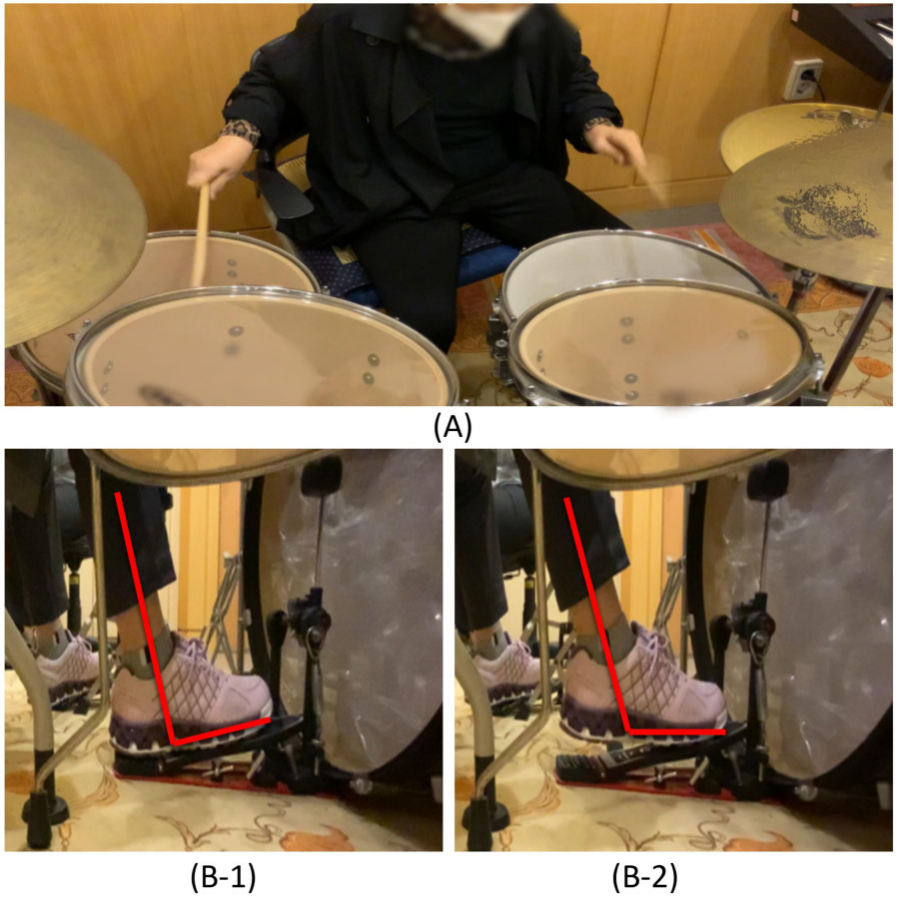
Examples of drum set playing used in the intervention. **(A)** Example of upper limb playing. **(B-1)** Example of lower limb playing using the heel-down position. **(B-2)** Example of lower limb playing using the heel-up position.

In the combined playing activity for interlimb coordination, the upper and lower limbs were activated simultaneously or alternately. Subdivision of pulse and manipulation of tempo in the musical material were used to grade the coordination demands and movement accuracy requirements according to the speed of upper and lower limb movement. The rhythmic playing aimed at continuous limb coordination was performed by complex rhythm patterns. This activity was performed using musical elements such as accents, dynamics, subdivisions of beat, and tempo, while adjusting playing intervals to induce continuous movements and support attention regulation. Additionally, at the end of each session, interactive playing with the therapist was recorded on a mobile phone and provided to the participant as visual and auditory feedback and to support motivation between sessions (Figure 2 for reference).

For the selection of music used in interventions, each participant provided information on their individual music preferences and daily music usage during a preliminary interview. Music with rhythms and tempos appropriate for drum set playing was selected, taking into consideration both familiarity and accessibility.

#### 2.5.3 Intervention considerations

The intervention was grounded in principles of dynamic systems theory, which emphasizes the role of task, environment, and individual constraints in shaping motor behavior. Structured musical tasks were designed to promote motor control through adaptive, goal-directed movement, with rhythmic elements serving both as temporal cues and as intrinsic motivators. The therapeutic use of music thus provided a dual function: reinforcing motor timing and sustaining participant engagement throughout the intervention.

**Figure 3:**
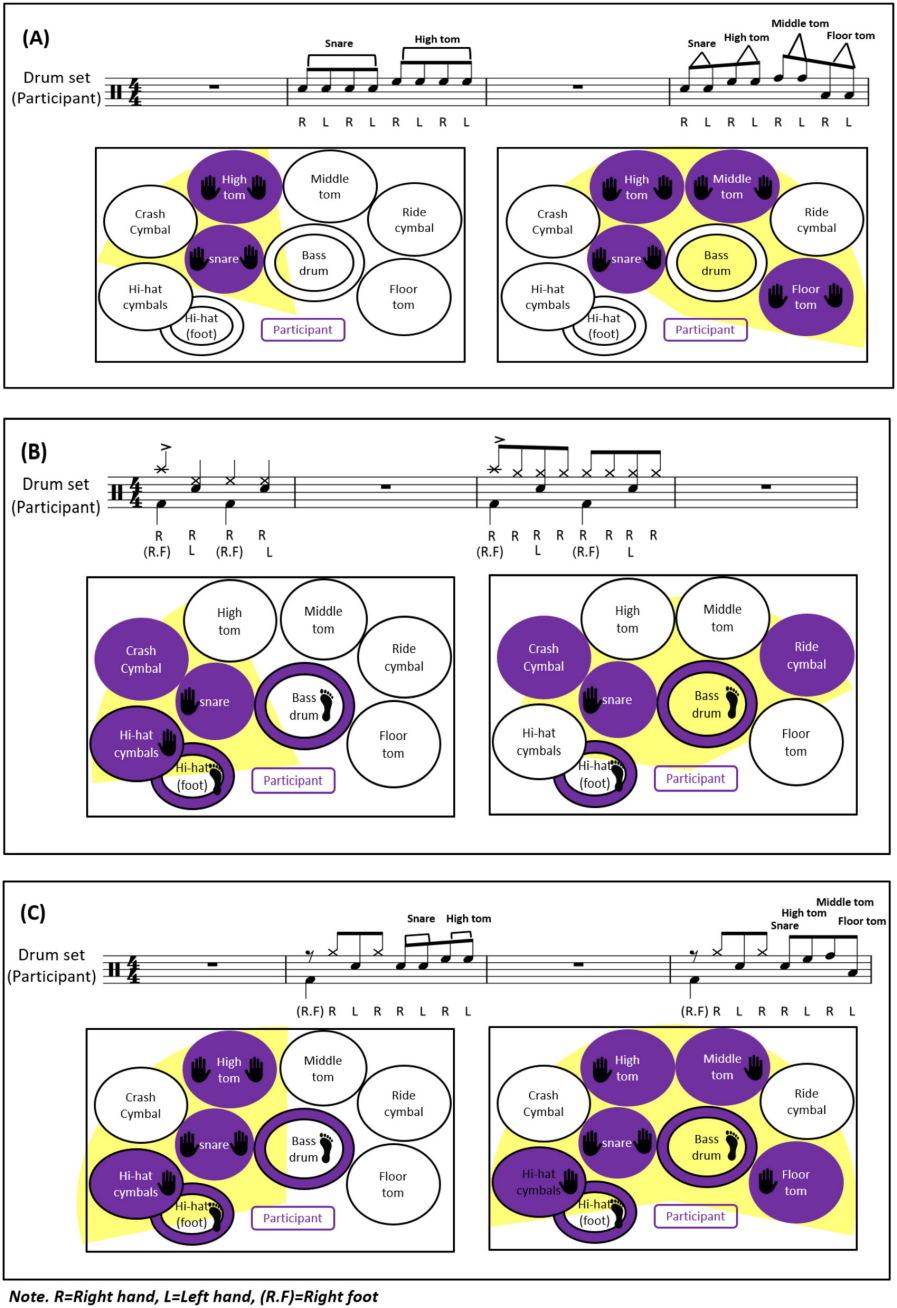
Examples of using the structural features of a drum set (purple color: the positions where the set drum is played, yellow color: range of motion when playing): **(A)** drum set playing alternating between hands; **(B)** drum set playing using both hands and the right foot; **(C)** drum set playing alternating both hands and foot. R, right hand; L, left hand; R.F, right foot.

First, to optimize motor learning in PD patients, the structure of the drum set was adjusted to maintain participant motivation by setting an appropriate level of difficulty. In the initial stage, participants practiced rhythm patterns at a comfortable pace to increase their familiarity with the sequence of movements involving alternating use of the upper and lower limbs. As the sessions progressed, the spatial arrangement of the instruments was adjusted to increase the range and intensity of movements. The drums were rearranged or combined in various configurations to facilitate new motor challenges, building on previously acquired movements (see Figure 3 for visual representation).

Secondly, structured auditory cues embedded within the musical framework were employed to assist participants in perceiving movement onset and regulating tempo-based actions. Previous studies have demonstrated that individuals with PD often experience difficulties in both initiating movement and maintaining sustained attentional focus (47, 48). To address these challenges, clearly defined auditory cues were provided during the early phases of intervention to enhance temporal anticipation and facilitate accurate movement initiation. In subsequent phases, consistent rhythmic cues at fixed tempi were introduced to support real-time motor adjustment and entrainment. Toward the final sessions, these cues were systematically faded to promote internalization of timing and encourage participants to independently initiate and regulate their movements without reliance on external auditory input.

## 3 Results

### 3.1 Observational results of individual session participation

#### 3.1.1 Participant A

At baseline, Participant A presented with a narrow step width, reduced arm swing during gait, and postural instability marked by lateral trunk lean when seated. During the preliminary interview, the participant reported frequent balance difficulties and instability during walking, as well as signs of cognitive decline. Based on this clinical profile, the intervention emphasized Stage 2 (separate playing) and Stage 3 (combined playing) of the protocol. In Stage 2, exercises targeted the upper and lower limbs independently to facilitate isolated coordination, incorporating musical elements such as accents and crescendo to modulate force output and encourage full arm extension with trunk rotation. To enhance ankle mobility, heel-down techniques were introduced, requiring foot alternation in response to tempo and accent changes. In Stage 3, simultaneous movements of ipsilateral upper and lower limbs were applied to promote interlimb coordination. The spatial arrangement of drums was designed to be progressively adjusted to challenge coordination in a stepwise manner.

At the beginning, Participant A had limited upper body control and frequently struck the edges of the high-tom and middle-tom drums instead of their centers. In addition, Participant A often had difficulty strongly or even slightly pressing the bass drum and hi-hat pedals, which require lower body movement. As the session progressed, the range of upper body movement expanded, and the accuracy of drum strikes and upper body stabilization while pressing the lower body pedals improved noticeably.

#### 3.1.2 Participant B

Participant B was observed to have slow walking speed, limited range of motion in the left shoulder and arm, and reduced range of motion in the ankle joint as the main problems. Although he had previously engaged in regular physical activities such as cycling, he reported that he had stopped exercising due to concerns about a recent fall and further loss of balance. The intervention focused on stages 3 (combined playing) and 4 (rhythmic playing), aiming to enhance simultaneous upper and lower body control and improve complex limb coordination. In stage 3, contralateral limb coordination training was conducted in addition to ipsilateral limb coordination. To this end, elements such as the spatial arrangement of the drums and the tempo of the music were adjusted to suit the participant's performance level. In stage 4, continuous rhythmic patterns were used to increase the temporal and spatial complexity of the previous motor skills.

In the initial sessions, Participant B exhibited insufficient arm extension and trunk leaning while alternating between tom drumming. In the ipsilateral limb task, it was difficult to move the lower limbs consistently. Over time, improvements were observed in trunk rotation and drumming accuracy, along with stabilized trunk control during limb movements. Playing time gradually increased, and the participant actively engaged in playing during tempo adjustments.

#### 3.1.3 Participant C

At the pre-intervention stage, Participant C displayed a forward-leaning gait, diminished arm swing, shortened step length, and reduced trunk rotation when turning. Visible tremors were observed in the left upper and lower limbs. During the initial interview, the participant reported difficulty using public transportation and recent initiation of cane use due to fear of falling. The intervention emphasized Stage 2 and Stage 3, targeting independent limb control and simultaneous upper-lower coordination. In Stage 2, drum movement parameters were adjusted to promote arm extension and trunk rotation, using musical features such as accents, crescendo, and decrescendo to support dynamic movement and force control. Heel-down and heel-up techniques were employed to improve joint mobility and stability across the lower limbs. Stage 3 introduced ipsilateral limb coordination with musical cues and drum spatial adjustments to support synchronized movement and modulation.

Initially, participant C exhibited excessive force when gripping the drumsticks due to tremors, which resulted in a dull drum sound and excessive compensatory movements when striking the floor tom drum located at the top. The bass drum performance was inconsistent and tilted to the right. As the session progressed, he demonstrated control over the force with which he gripped the drumsticks, and his footwork improved to the point where he was able to play the bass drum more clearly, resulting in a more stable performance overall.

### 3.2 Changes in motor control

**Figure 4:**
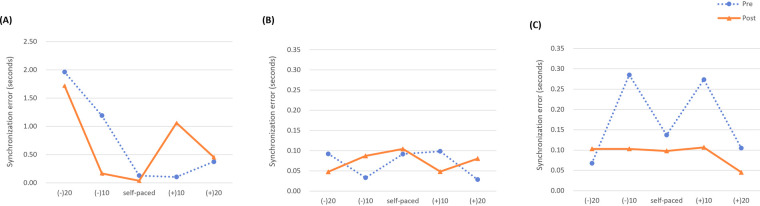
Changes in synchronization errors for the bimanual drum tapping task. The mean synchronization errors during bimanual tapping according to the five conditions (self-paced, +10% tempo, +20% tempo, −10% tempo, and −20% tempo) were measured pre- and post-intervention for each participant. **(A)** Participant A, **(B)** Participant B, **(C)** Participant C.

To assess changes in participants' motor control following the intervention, assessments of balance (TUG, BBS), upper limb function (BBT, 9-HPT), upper limb control (MIDI-drum tapping task), and cognitive function (TMT, K-CWST) were conducted. The pre- and post-test results for each participant are presented below (refer to Table 3 and Figure 4).

**Table 3:** Results of balance, upper limb function, and cognitive evaluation.

Participants		TUG (seconds)	BBS	BBT		9-HPT (seconds)		TMT (seconds)		K-CWST (seconds)	
				R	L	R	L	Part A	Part B	Word reading	Color reading
A	Pre-test	22.36	27	13	14	50.90	56.02	40.81	80.25	138.05 (3 errors)	124.13 (66 errors)
	Post-test	21.47	30	26	24	41.40	59.97	27.49	96.26	123.46 (2 errors)	291.97 (40 errors)
	Change	−0.89	3	13	10	−9.5	3.95	−13.32	16.01	−14.59 (−1 error)	167.84 (−26 errors)
B	Pre-test	12.40	55	35	45	27.89	36.90	17.59	21.68	67.77 (no error)	107.33 (2 errors)
	Post-test	10.53	56	39	37	26.15	28.25	14.63	21.75	74.49 (no error)	110.73 (no error)
	Change	−1.87	1	4	−8	−1.74	−8.65	−2.96	0.07	6.72 (no error)	3.4 (−2 errors)
C	Pre-test	14.81	47	22	25	47.52	46.25	24.38	38.25	84.90 (3 errors)	219.76 (38 errors)
	Post-test	13.55	49	44	35	39.05	34.97	23.78	38.82	74.77 (no errors)	217.46 (19 errors)
	Change	−1.26	2	22	10	−8.47	−11.28	−0.6	0.57	−10.13 (−3 errors)	−2.3 (−19 errors)

BBS, Berg Balance Scale; BBT, Box and Block Test; K-CWST, Korean-Color Word Stroop Test; 9-HPT, 9-Hole Peg Test; TMT, Trail Making Test; TUG, Timed Up and Go test.

Participant A showed a decrease in TUG time (−0.89 s) and an increase BBS score (+3). In the upper limb function assessments, BBT scores increased (right = +13; left = +10), and 9-HPT completion time decreased for the right hand (−9.5 s) and increased for the left hand (+3.95 s). In the upper limb control assessment (MIDI-drum tapping), Participant A's tapping baseline (self-paced) was measured at 121 bpm. The average synchronization error decreased at self-paced, −20%, and −10% tempos, and did not change at +10% and +20% tempos. In the cognitive assessments, TMT completion time changed by −12.32 s for type A and +16.01 s for type B. On the K-CWST, word condition time changed by −14.59 s (errors −1), and color condition time changed by +167.84 s (errors −26).

Participant B showed a decrease in TUG time (−1.87 s) and an increase in BBS score (+1). In the upper limb function assessments, BBT scores changed by +4 (right) and −8 (left), and 9-HPT completion time changed by −1.74 s (right) and −8.65 s (left). In the upper limb control assessment (MIDI-drum tapping), Participant B's tapping baseline (self-paced) was measured at 130 bpm. The average synchronization error decreased at −20% and +10% tempos and did not change at self-paced, −10%, or +20% tempos. In the cognitive assessments, TMT completion time changed by −2.96 s for type A and +0.07 s for type B, and K-CWST word and color condition times changed by +6.72 s (errors 0) and +3.4 s (errors −2), respectively.

**Figure 5:**
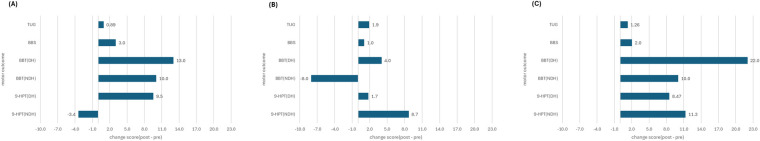
Participant-wise change scores for key motor outcomes following the BEAT program. Bars represent post–pre change scores that were improvement-coded so that positive values indicate improvement for all measures. For BBS and BBT, higher scores reflect better performance and are shown with their original sign; for TUG and 9-HPT, change scores were sign-reversed so that decreases in completion time appear as positive values. **(A)** Participant A, **(B)** Participant B, **(C)** Participant C.

Participant C showed a decrease in TUG time (−1.26 s) and an increase in BBS score (+2). In the upper limb function assessments, BBT scores increased (right = +22; left = +10), and 9-HPT completion time decreased for both hands (right = −8.47 s; left = −11.28 s). In the upper limb control assessment (MIDI-drum tapping), Participant C's tapping baseline (self-paced) was measured at 118 bpm. The average synchronization error decreased at self-paced (118 bpm), +10%, +20%, and −10% tempos. In the cognitive assessments, TMT completion time changed by −0.6 s for type A and +0.57 s for type B, and K-CWST word and color condition times changed by −10.13 s (errors −3) and −2.3 s (errors −19), respectively. Individual change profiles for motor outcomes (TUG, BBS, BBT, and 9-HPT) are illustrated in Figure 5 and Table 4.

**Table 4:** Pre–post change scores and exploratory effect sizes (Cohen's *d*_pre).

Body region	Motor outcome measure	Hand	Pre (*M* ± SD)	Post (*M* ± SD)	MD (Post–Pre, *M* ± SD)	Cohen's *d*_pre
Low limb	BBS		43.00 ± 14.42	45.00 ± 13.45	2.00 ± 1.00	0.14
	TUG		16.52 ± 5.19	15.18 ± 5.65	−1.34 ± 0.49	−0.26
Upper limb	BBT	Dominant	23.33 ± 11.06	36.33 ± 9.29	13.00 ± 9.00	1.18
		Non-dominant	28.00 ± 15.72	32.00 ± 7.00	4.00 ± 10.39	0.25
	9-HPT	Dominant	42.10 ± 12.42	35.53 ± 8.21	−6.57 ± 4.21	−0.53
		Non-dominant	46.39 ± 9.56	41.06 ± 16.71	−5.33 ± 8.14	−0.56

MD represents post–pre change scores. Positive MD indicates improvement for BBS and BBT, whereas negative MD indicates improvement for time-based measures (TUG, 9-HPT). Cohen's *d*_pre was calculated as (*M*_post -*M*_pre)/SD_pre; values of ∼0.2, ∼0.5, and ≥0.8 are commonly interpreted as small, medium, and large effects, respectively. Effect sizes are reported descriptively and should be interpreted cautiously given the pilot sample size (*n* = 3).

### 3.3 Changes in depression and quality of life

To assess changes in emotion and quality of life following the intervention, K-GDS and PDQ-39 were completed by the participants before and after the intervention. For Participant A the K-GDS score increased from 13 to 15 and the PDQ-39 total score decreased from 33.3 to 16. For Participant B, the K-GDS score increased from 11 to 14 and the PDQ-39 total score increased from 24.3 to 26.2. For Participant C, the K-GDS score decreased from 13 to 12 and the PDQ-39 total score decreased from 29.4 to 23.7. The pre- and post-test results for each participant are presented in Table 5.

**Table 5:** Results of K-GDS and PDQ-39 in participants.

Participant		K-GDS (/30)	PDQ-39 (/100)
A	Pre-test	13	33.3
	Post-test	15	16
	Change	+2	−17.3
B	Pre-test	11	24.3
	Post-test	14	26.2
	Change	+3	+1.9
C	Pre-test	13	29.4
	Post-test	12	23.7
	Change	−1	−5.7

K-GDS, Korean-Geriatric Depression Scale; PDQ-39, Parkinson's Disease Questionnaire, higher number = worse PDQ.

### 3.4 Participants' post interview results

#### 3.4.1 Theme 1: engagement and exploration—initiating musical risk and autonomy

All three participants described the experience of engaging with drum set playing as novel, challenging, and motivating. Despite concerns related to their physical and cognitive limitations, each demonstrated a strong willingness to participate and initiate musical activity often for the first time.

Participant A, who expressed initial hesitation about his physical capacity, noted: “I’m worried if I can play the drum set because I have Parkinson's disease. But I really want to try. Do you think I can do it? If you say I can, I’d love to give it a shot.”

Participant B, who had no prior musical experience, shared: “I was interested in playing the drum set, so I applied to participate in a research study for the first time. But I’m not sure if I’ll be able to play well.”

Participant C also emphasized her proactive effort to prepare: “I had never seen a study involving drum performance before. I really wanted to participate, so I applied, but I was worried about whether I’d be able to play. I searched the internet and looked up videos on YouTube.”

The participants' narratives reflected their serious and active participation in drumming. Despite uncertainty about the new experience of playing a drum set, all participants actively participated in the playing process. This behavioral change indicates that the musical performance outcomes experienced in this intervention provided individuals with new auditory aesthetic stimuli, which led to motivation.

#### 3.4.2 Theme 2: perceived change—physical, emotional, and social dimensions

The interview data showed changes in perception related to various functional areas. Participants consistently reported improvements in their physical perceptions, such as limb control and confidence in movement. Emotionally, they experienced motivation and enjoyment through the rhythmic music performance process, despite initial anxiety about the new challenge and frustration at not being able to move as they wanted.

Participant A emphasized the feasibility of seated drumming and the new coordination demands: “When asked to do other therapies standing up, I felt like I might fall over… But sitting down and doing this was okay. I could lift my feet, and I could keep doing it. I hadn't tried moving my hands and feet together like this before. When I thought about it, I realized it might help me.”

Participant B described an increase in lower limb control and self-efficacy: “I think I’ve started to develop more strength in my foot… I feel more confident—I feel like I want to try other things, as if I can do anything. I showed a video of myself playing to my daughter. She told me I was doing well, and we laughed together when I made a mistake.”

Participant C expressed how full-body involvement enhanced both cognitive motivation and mood: “Playing the drum set involves moving the whole body… it feels like exercise. I think it might even help prevent dementia… It lifts my mood—refreshing? Motivation! It gives me a lot of motivation. I’d really recommend it to others… Coming here makes my whole day enjoyable—it gives me energy and a sense of purpose.”

Participants’ reports showed changes in both physical and psychosocial dimensions. It was clear that playing an instrument was meaningful, and physical participation in particular was meaningful. It was also clear that encouragement and expectations from family and others served as positive external feedback. These factors can serve as valuable resources for supporting continued participation.

#### 3.4.3 Theme 3: musical connection—familiarity, enjoyment, and emotional resonance

Participants noted that the use of familiar or personally meaningful music during sessions enhanced their sense of engagement and enjoyment. Music that held cultural or emotional significance was described as contributing to positive affect and appeared to support sustained participation in the intervention.

Participant A noted the emotional activation linked to a familiar song: “The song I played was one everyone knows. It's a song I used to sing when I was young—I really liked singing. Even now, when that song comes on at home, I find myself moving my hands and feet along with it.”

Participant B reflected on the motivational effect of using known repertoire: “It felt good to use familiar music… I wanted to play the entire song… I honestly loved everything. It wasn't hard at all.”

Participant C, who actively sought related material outside of the session, shared: “I enjoyed participating because the music used in the sessions included songs I personally liked. At home, I also looked up drum performance videos and music related to the sessions.”

This theme illustrates how music selection shaped the emotional and motivational environment of the intervention. When familiar songs were used, participants reported increased emotional engagement and extended their musical curiosity beyond the sessions, reflecting high levels of intrinsic motivation.

## 4 Discussion

This case study presents a pilot investigation into the potential of structured drum set playing, operationalized as the BEAT program, as an integrative therapeutic intervention for individuals with PD. In contrast to conventional music therapy practices that often rely on simple, repetitive bimanual tasks involving a single percussion instrument, the BEAT program incorporates full drum set performance tasks that require coordinated engagement of all four limbs. By emphasizing bilateral integration and rhythmic entrainment through complex motor sequences, this approach was designed to provide functional benefits across motor, cognitive, and psychosocial domains. Given the small sample size and uncontrolled design, the present findings should be regarded as preliminary and hypothesis generating rather than confirmatory. Based on the outcomes of the pilot intervention, the following key findings are discussed.

One of the primary findings of this study was an observable pattern of change in lower limb coordination and dynamic balance following participation in the BEAT program. Post-intervention assessments revealed a mean reduction in Timed Up and Go (TUG) times and an increase in Berg Balance Scale (BBS) scores, suggesting enhanced motor control in ambulatory tasks. These gains may be related to performance parameters embedded in the program—particularly pulse subdivision, accent variation, and tempo changes. Subdivision patterns encouraged alternating foot movement, facilitating dorsiflexion and plantar flexion cycles critical for gait, while accented heel-up playing activated musculature associated with ankle stability (49). Tempo modulation may have further supported temporal regulation of coordinated upper and lower limb activity (22). At the individual level, all three participants showed pre-post changes on TUG and BBS in the direction of improved performance, although the magnitude of change differed across cases. These results are descriptively consistent with prior studies on rhythm-based motor rehabilitation in PD (50), and illustrate the potential functional applicability of the BEAT program in addressing lower limb motor deficits.

Enhancement in upper limb dexterity also emerged as a descriptive outcome of the BEAT intervention. Fine and gross motor functions, assessed through the Nine-Hole Peg Test (9-HPT) and Box and Block Test (BBT), showed pre-post changes consistent with improved performance in most, but not all, limbs. These changes may have been facilitated by the complex sensorimotor requirements of drumming particularly the integration of grip modulation, bilateral hand alternation, and graded force control through dynamics and rhythmic variation. Musical cues such as crescendo and decrescendo demanded real-time adjustments in amplitude and spatial range, recruiting muscle groups across the wrist, elbow, and shoulder (51). However, the degree of change varied by participant, with individualized patterns that appeared to be linked to pre-existing functional asymmetries and differing levels of task engagement. These exploratory findings highlight the potential importance of tailoring rhythmic motor interventions to individual profiles, in line with existing literature on personalized rehabilitation in PD (52, 53). To complement the descriptive pre–post findings, exploratory effect sizes (Cohen's *d*_pre) were calculated to estimate the magnitude of change relative to baseline variability. These values suggested heterogeneous change profiles across outcomes. Balance and functional mobility measures showed small effects (BBS *d*_pre = 0.14; TUG *d*_pre = −0.26), indicating modest improvements in overall postural control and gait performance. In contrast, upper-limb outcomes demonstrated larger magnitudes of change, particularly for right-hand gross dexterity (BBT-Right *d*_pre = 1.18) and bilateral fine motor speed (9-HPT-Right *d*_pre = −0.53; 9-HPT-Left *d*_pre = −0.56), reflecting moderate-to-large reductions in task completion time. Left-hand BBT changes were smaller (*d*_pre = 0.25), likely influenced by individual asymmetries and mixed directional changes across cases. Importantly, given the pilot case-series design and very small sample size (*n* = 3), these effect-size estimates are intended as preliminary, hypothesis-generating indicators and should be interpreted with substantial caution rather than as evidence of efficacy.

The BEAT program also appeared to be associated with small changes in upper limb motor control and cognitive-motor integration. MIDI-based tapping tasks revealed reduced synchronization errors, particularly under tempo-reduction conditions that required higher cognitive demand. These outcomes may reflect modest improvements in timing prediction, attention maintenance, and executive processing—faculties essential for rhythm-based coordination (54). Structured elements of the musical tasks, such as planned rests and dynamic tempo changes, may have facilitated initiation and inhibition processes, while auditory cues functioned as external timekeepers to guide performance execution (37, 59). Additionally, participants who appeared to engage more actively with tempo-variant tasks showed relatively greater stability in cognitive test performance, although these patterns must be interpreted cautiously in light of the very small sample. Taken together, these observations support the notion that complex motor control emerges from sensorimotor-cognitive interaction, rather than motor execution alone (7, 60).

While quantitative assessments of quality of life produced mixed results, participants consistently reported subjective emotional benefits from engaging in the BEAT program. Interviews revealed increases in daily vitality, emotional expression, and confidence in taking on new challenges. These psychosocial shifts may be attributed to the immediate sense of accomplishment and self-efficacy evoked by successfully coordinating complex rhythm patterns. However, standardized tools such as the K-GDS and PDQ-39 reflected small and inconsistent changes across participants. For example, only one participant showed a measurable decrease in depressive symptoms, while others showed either no change or worsening scores, despite positive qualitative feedback. In addition, improvements in PDQ-39 were not uniform, with one participant showing a reduction in total score and another showing a slight increase. This divergence underscores both the importance of individualized contextual factors (e.g., age, symptom severity, social environment) and the limitations of existing instruments in capturing subtle affective and psychological changes (55, 56). It is also possible that expectancy effects or social desirability biases influenced self-report ratings, which may have contributed to the discrepancy between the largely positive interview narratives and the small or negative changes observed on K-GDS and PDQ-39. Given the brief, low-dose nature of the intervention (30-minute sessions, one to two times per week for 6 weeks), the absence of robust and consistent changes in depression and quality-of-life scores is not unexpected. Longer-term and more intensive interventions may be required to consolidate emotional outcomes in daily functioning (57, 58).

This study has several limitations that must be acknowledged. First, the findings are based on a small sample of three community-dwelling individuals with Parkinson's disease, which limits the generalizability of the results to broader populations. Second, due to participants' advanced age and individual circumstances—including fluctuating physical condition, medical appointments, and transportation constraints—it was not feasible to conduct the intervention on consistent days or times. Consequently, the BEAT program was delivered once or twice per week across ten sessions, with scheduling flexibly adjusted to accommodate each participant. This relatively low and variable intervention does may have constrained the magnitude and stability of observable changes. Third, no follow-up assessments were conducted, so it is unknown whether any observed changes were retained over time or generalized to everyday activities. Finally, the use of a case study design with descriptive analyses and no control group precludes causal inference. To more rigorously evaluate the efficacy of the BEAT program and estimate its effects, larger-scale studies employing randomized controlled designs and longer follow-up periods are necessary.

## 5 Conclusion

In conclusion, this pilot investigation provides preliminary hypothesis-generating observations suggesting that the BEAT program—Bilateral Engagement through Active Drumming in Music Therapy—may be a feasible and engaging intervention that could support improvements in motor control, interlimb coordination, and subjective quality of life among individuals with Parkinson's disease. Beyond physical outcomes, observed trends also suggest the possibility of benefits for cognitive-motor integration and emotional well-being in this small, uncontrolled sample. Further research is warranted to validate these findings, explore the program's effects on higher-order cognitive functions such as response inhibition and attentional flexibility, and optimize intervention delivery based on individualized clinical profiles. The BEAT program represents a novel and functionally oriented conceptual therapeutic model that integrates rhythmic entrainment and bilateral motor activation within a music therapy framework, with potential applicability to neurorehabilitation that should be examined in larger controlled trials.

## Data Availability

The original contributions presented in the study are included in the article/Supplementary Material, further inquiries can be directed to the corresponding author.
